# A Case of Ectopic Spleen Transplanted Into the Sigmoid Colon and Pelvic Peritoneum

**DOI:** 10.1155/carm/4875687

**Published:** 2025-08-29

**Authors:** Ming Liu, Miao Zhang, Jingyu Xiang, Yifang Zhang, Jian Liu

**Affiliations:** Department of Gynecologic Oncology, First Affiliated Hospital, Bengbu Medical University, Bengbu 233004, Anhui, China

**Keywords:** case report, ectopic spleen, pelvic mass, pelvic peritoneum, sigmoid colon

## Abstract

Ectopic spleen (ES) is a rare complication of autologous transplant following spleen injury. Autopsy studies suggest that the true incidence rate could be as high as 67%, though only 5%–10% of patients require clinical intervention. This case report describes a highly cautionary case of ES, with an extremely long latent period. The patient presented with dull lower abdominal pain 24 years after undergoing a splenectomy. An ultrasound examination revealed a round-shaped, solid mass measuring 1.6 × 2.5 cm adjacent to the right ovary. Following laparoscopic surgery, a 2.5 × 1.5 cm mass on the surface of the sigmoid colon and a 1.0 cm dark red nodule on the pelvic floor were completely removed. Pathological and immunohistochemical examinations confirmed that both lesions were splenic tissue. This case highlights the fact that the latent period of ES can last for decades. For patients with a history of splenectomy, any new pelvic or abdominal masses should be included in the differential diagnosis of ES. We urge the implementation of standardized long-term follow-up mechanisms combined with multimodal imaging techniques for a comprehensive assessment in order to effectively avoid misdiagnosis and overtreatment.

## 1. Introduction

Ectopic spleen (ES) is a rare phenomenon in autologous tissue transplantation. The mechanism involves the dispersion and implantation of splenic parenchymal cells in the abdominal area after spleen injury or surgery [1]. Autopsy studies have shown that the incidence of ES can be as high as 67%, but only 5%–10% require clinical intervention due to the placeholder effect or suspicion of malignancy [2]. It is worth noting that the pelvis is a rare target area for ES, and its anatomical peculiarities often result in overlapping lesions with the imaging characteristics of gynecologic tumors, leading to overtreatment [3]. This article reports a case of sigmoid–pelvic peritoneum complex ES after splenectomy with an extremely long incubation period of 24 years and reviews the relevant literature.

## 2. Case Report

**Figure 1:**
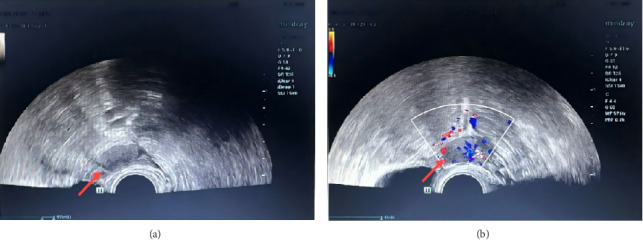
The ultrasound shows a solid, hypoechoic mass that is intimately attached to the outer border of the right ovary and has abundant blood flow signals.

**Figure 2:**
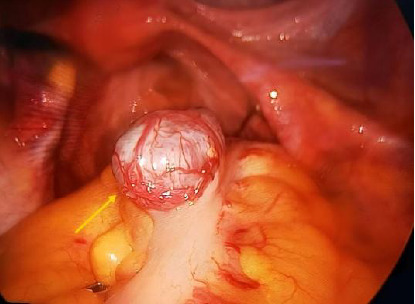
Ectopic splenic tissue found during the procedure in the sigmoid colon.

**Figure 3:**
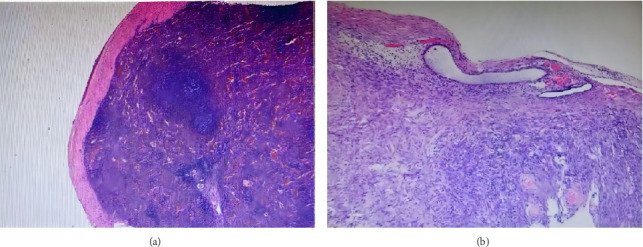
Histopathology (hematoxylin and eosin stain). (a) A sigmoid colon mass showed blood vessels and lymph nodes microscopically, along with significant hemorrhage in the red pulp region. Immunohistochemical marker results showed CD3 (1+, T cells), CD3 (3+, blood vessels), and CD20 (2+, B cells), consistent with the splenic tissue. (b) The pelvic peritoneal nodules consist of congested splenic tissue.

A 50-year-old woman was admitted to the hospital with dull pain in the lower abdomen for 2 months. Physical examination: A firm mass approximately 3 cm in diameter was palpated in the right accessory region, with good mobility and no tenderness. Laboratory studies showed no significant abnormalities in routine blood tests, liver and kidney function, tumor markers, etc. An ultrasound scan revealed a hypoechoic lesion measuring 1.6 × 2.5 cm adjacent to the right ovary (Figure 1), with abundant blood flow. No enlarged abdominal lymph nodes, fluid accumulation, or other abnormal structures were observed. Although PET-CT has certain advantages in distinguishing between benign and malignant masses, the mass in this case was located deep in the pelvis and adjacent to the intestinal tract. This limited the spatial resolution and specificity of PET-CT in this anatomical region. Furthermore, the patient's clinical presentation and conventional imaging studies did not indicate any obvious malignant features. Taking into account the patient's financial situation and the local medical resources available, PET-CT was not performed. Past medical history: 24 years ago, an open splenectomy was performed due to a ruptured spleen caused by a car accident. The intraoperative record shows that a large amount of saline was used to irrigate the abdominal cavity. Due to persistent abdominal pain and an unclear pelvic mass, it was not possible to rule out malignant tumors entirely. Furthermore, there was a potential risk of torsion or bleeding from the mass. Therefore, a diagnostic laparoscopy was ultimately decided upon. The surgical procedure was performed under general anesthesia, and the approach adopted was a four-port laparoscopic one. A 2.5 × 1.5 cm solid mass was found on the surface of the sigmoid colon (Figure 2). The mass had penetrated the intestinal wall and had a rich supply of surface blood vessels, with no adhesion to the surrounding area. Another dark red nodule measuring 1.0 cm was found on the right pelvic peritoneum. The lesion was completely removed using a monopolar electrosurgical hook. The surgery was successful, with only around 50 mL of blood lost during the procedure, and there was no need to convert to open surgery. The patient was discharged from the hospital four days after surgery and made a full recovery. A follow-up examination three months later revealed no abdominal pain or other discomfort. Pathological and immunohistochemical examinations showed that both lesions consisted of spleen tissue (Figure 3). According to the patient's recollection, she did not receive the pneumococcal, hemophilus influenzae Type b (Hib) and meningococcal vaccines after splenectomy 24 years ago. All ES tissue was removed during the operation. Following discharge, the patient received the above vaccines and was provided with individualized health education and regular follow-up to reduce the risk of severe infection.

## 3. Discussion

ES is relatively rare in clinical practice and usually has no obvious symptoms. Studies have shown that there is significant individual variation in the average time from spleen injury to diagnosis of ES (5 months–32 years) [4]. This may be closely related to the location of ES tissue implantation, growth rate, and host immune regulatory mechanisms. In this case, the interval from splenectomy to ES diagnosis was 24 years, consistent with the upper limit reported in the literature. This is an indication that long-term follow-up of patients after splenectomy should be vigilant.

Residual splenic tissue has a strong regenerative potential, and its proliferation process may be coregulated by the local microenvironment and circulating growth factors [5]. Currently, the mechanism of ES formation mainly involves three pathophysiological processes [6]: (1) dissemination of splenic tissue fragments after trauma, (2) implantation caused by abdominal cavity lavage during splenectomy, and (3) splenic myeloid cells dissemination via the circulation. Different mechanisms of origin may lead to differences in the anatomical distribution of ES. Pelvic ES is more likely to be associated with the first two mechanisms, whereas intrahepatic lesions are more likely to result from hematogenous metastasis.

Pelvic ES may be confused with gynecologic diseases (such as endometriosis and ovarian tumors) [7], while lesions in other parts of the abdominal cavity must be differentiated from metastatic tumors or lymphoproliferative diseases. In imaging characteristics, uniform enhancement during the arterial phase of CT (synchronized with the spleen) and MRI, showing slightly low signal intensity with T2-weighting, along with signal attenuation in the antiphase, remains significant for distinguishing ES from well-vascularized tumors (e.g., neuroendocrine tumors or hemangiomas) [8, 9]. Despite the specificity of 99mTc DRBC nuclear scans, their sensitivity is limited by the size of the lesion (significantly reduced for diameters less than 2 cm) and the functional status of the splenic tissue [10, 11]. Ultrasound/CT-guided puncture biopsy can theoretically provide a basis for diagnosis, but in practice, it is often limited by factors such as the rich blood supply of the lesion and difficulty in positioning. Recent studies have suggested that, despite the heterogeneity of contrast-enhanced ultrasound (CEUS) arterial phase manifestations, the persistent enhancement features observed in the portal and delayed phases demonstrate a high degree of consistency with those of CT/MRI [12]. This consistency suggests that CEUS can serve as a reliable diagnostic tool for ES, particularly in scenarios where CT/MRI is not accessible.

PET-CT is a functional imaging technique that demonstrates high sensitivity and specificity in distinguishing between benign and malignant masses, staging tumors, and detecting distant metastases. It is widely used in the diagnosis and management of various malignant tumors. However, its spatial resolution and specificity for deep pelvic masses are limited, particularly in anatomically complex regions such as those adjacent to the intestines. Furthermore, it is prone to false-positive results, influenced by inflammation and nontumor factors. In this case, the patient's clinical presentation and conventional imaging studies did not suggest significant malignant features. Given the patient's financial situation and the limited availability of medical resources locally, a PET-CT was not performed. The diagnosis was primarily based on ultrasound findings and laparoscopic surgical pathology results. This case highlights the importance of making an informed decision about which imaging studies to use in clinical practice, taking into account the patient's specific circumstances.

In this case, due to the inadequate utilization of imaging methodologies, laparoscopic surgery was ultimately necessitated for the purpose of obtaining a pathological diagnosis. This case indicates that advancing multimodal imaging technology is crucial for preventing overtreatment. It is particularly important to note that although ES tissue possesses certain immune defense functions, its immune protective efficacy is significantly lower than that of a normal, intact spleen. Patients remain at long-term risk of severe infections (such as postsplenectomy sepsis) following splenectomy. Therefore, infection prevention and control measures, as well as standardized follow-up care, should not be overlooked in clinical management.

## 4. Conclusion

ES cells retain partial spleen function and grow slowly, with a latency period that can span several decades. In patients with a history of splenectomy, any new pelvic/abdominal mass should be included in the differential diagnosis. It is recommended to establish a long-term follow-up mechanism [13] and conduct comprehensive assessments using multimodal imaging (CT, MRI, CEUS, etc.) to avoid misdiagnosis and overtreatment due to insufficient knowledge. Surgical intervention is indicated in the following situations: (1) clinical symptoms due to mass effect, (2) imaging findings that are unable to exclude the presence of malignant tumors, and (3) the occurrence of complications such as torsion or bleeding. Laparoscopic exploration is considered both a safe and effective diagnostic method and the preferred treatment option, particularly when imaging studies are inconclusive. Clinicians should enhance their awareness of ES, particularly in patients with a history of splenectomy, and thoroughly consider the possibility of ES to reduce diagnostic delays and avoid unnecessary surgical risks. In addition, all patients with a history of splenectomy or ectopic splenectomy are recommended to adhere to international and local guidelines for completing pneumococcal, *Neisseria gonorrhoeae*, Hib, and other pathogen vaccinations. Based on individual risk factors, regular infection risk assessments and follow-ups should be conducted. Health education for patients and their families should also be strengthened to minimize complications resulting from impaired immune function.
